# Supplementary figures and images for: Role of the repeat expansion size in predicting age of onset and severity in RFC1 disease
Source: Brain. 2024 Jan 9;147(5):1887–98. doi: 10.1093/brain/awad436 (PMC11068103; doi:10.1093/brain/awad436)

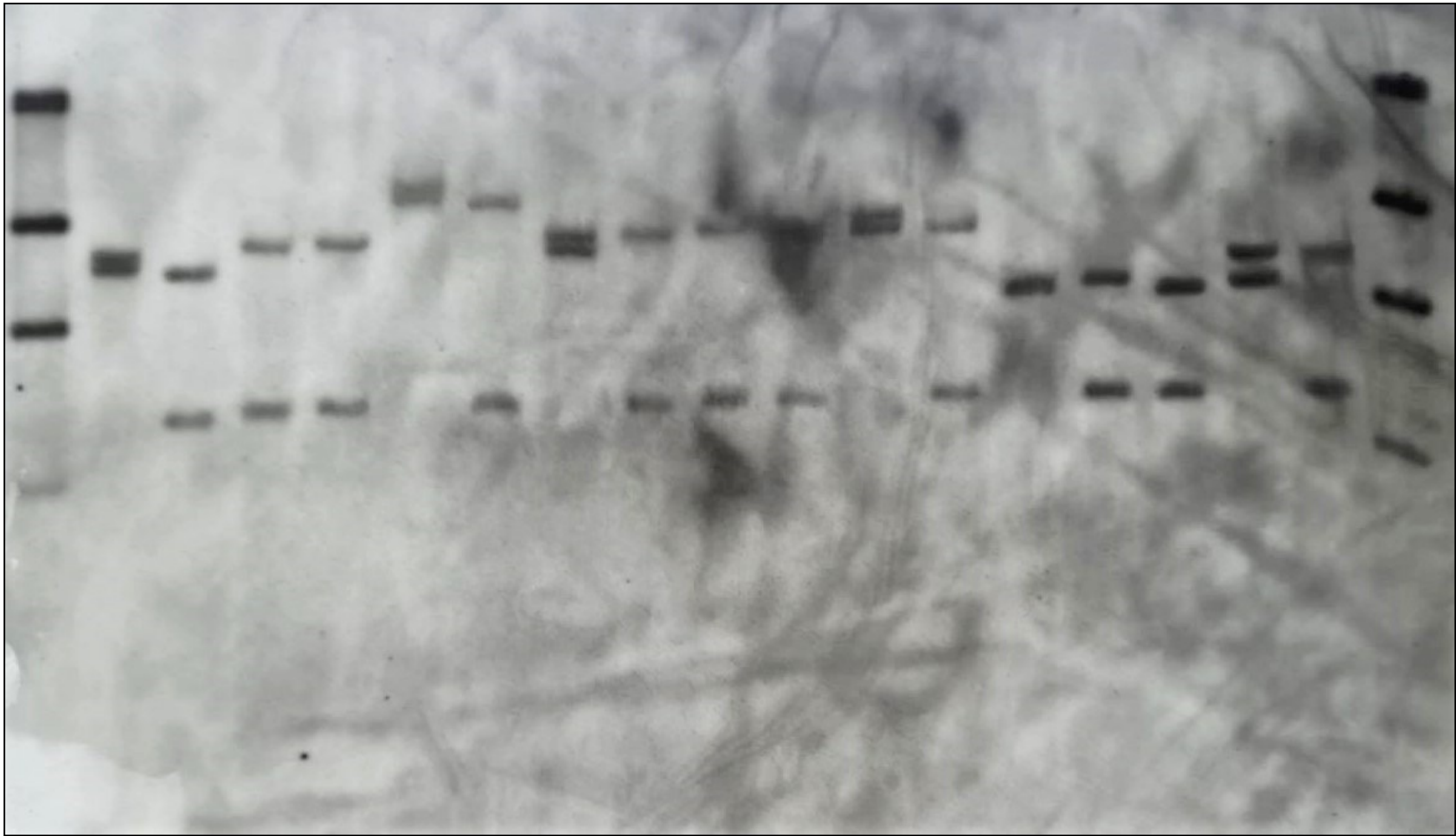

Supplement: awad436_Supplementary_Data [file awad436_supplementary_data.zip › brain-2023-02058-File008.pdf]
